# Standardization of Clinical Photos for Tracking Management of Hair Loss in Dermatology Clinics

**DOI:** 10.1111/jocd.70381

**Published:** 2025-08-07

**Authors:** Lucy Rose, Aliza Khuhro, Samantha Rojas, Stephanie Adame, Matthew Gallardo, Karissa Libson, Trent Walker, Brittany Dulmage

**Affiliations:** ^1^ The Ohio State University College of Medicine Columbus Ohio USA; ^2^ The Department of Dermatology College of Medicine, The Ohio State University Wexner Medical Center Columbus Ohio USA

**Keywords:** alopecia, hair loss, quality improvement

## Abstract

**Background:**

Despite its usefulness, clinical photos are not frequently utilized for hair loss patients seen in dermatology due to inefficiency and lack of standardization. Standardized photography for hair loss patients has been shown to improve patient motivation, satisfaction, and treatment monitoring.

**Aims:**

This study introduced standardized photography protocols through educational materials for staff with the aim of improving clinical documentation for hair loss patients.

**Methods:**

Over an initial 10‐month period, 32% (16/50) of randomly selected patients presenting with a chief complaint of hair loss to an academic dermatology practice had optimal standardized photos. Though numerous underlying factors likely accounted for this shortcoming, one contributing cause was that nurses and medical assistants (MAs) in the department were not trained to obtain necessary photos for each visit. We aimed to increase correct standardized photos in a dermatology clinic to 60% of hair loss patients. We created a 2‐min video demonstrating three main points: when to take hair loss photos during appointments, which views to take, and how to upload pictures into patient charts. This video was distributed to all nurses and MAs in the department.

**Results:**

After the distribution of the training video, nurses and MAs were statistically significantly more likely to take the correct hair loss photos, with 66% (33/50) of patients having correct pictures taken with a minimum of three views (*p* < 0.005).

**Conclusions:**

Standardized photography for hair loss patients is a simple intervention with positive benefits. By improving clinical documentation, care, and outcomes for patients seeking treatment for hair loss can be improved.

## 1 Introduction

While there are many treatments for hair loss, the nature of the hair cycle timeline paired with the desired short turnaround time for results leaves high attrition rates among patients with hair loss [1]. Additionally, the progression of hair loss conditions can be slow, often with noticeable results taking longer than 6 months, making it difficult for patients to track subtle changes between appointments [2]. A retrospective cohort study found that photographic assessment of hair has been shown to improve follow‐up rates in patients with androgenic alopecia and alopecia areata [1]. Photos allow both physicians and patients to identify improvement, as prior work has demonstrated that patients are able to compare therapeutic effects when shown photos [3]. Additionally, photos increased patient satisfaction, decreased complaints, and improved tolerability for procedures [3]. Research from Pathoulas et al. shows standardized photography among dermatology patients with hair loss decreases anxiety, leaves patients feeling more motivated to follow through with treatment, and benefits patient outcomes [4]. These studies suggest that medical observation, in the form of photographic assessment, can help patients feel more motivated as they continue with therapy and follow‐up visits.

Hair loss can be diagnosed and assessed with a variety of tests, including biopsies, trichoscopy, visual classification systems, and patient questionnaires [5]. Photographic assessment of hair is recognized as a useful tool in documenting patient progress, but has been noted to be cumbersome, time‐intensive, and neither standardized nor optimized for best patient outcomes in modern clinic workflow [6]. Some challenges for regular use of photographs in clinic include minimal training with department cameras or mobile phones and lack of consistency in the photographic views and image quality (i.e., lighting, location), which diminish the value of these images in longitudinal assessment of patients [5]. However, the value of clinical photography in dermatology is immense, and it allows for improved monitoring of hair loss, improved patient understanding of hair loss conditions, and improved communication between patient and provider [7]. This highlights a need for standardized, user‐friendly, and efficient protocols in hair loss photography that can improve patient care and outcomes in clinical practice.

Before the era of smartphones, clinical photographs were largely developed on film, necessitating that the storage of these photos be tangible with lock and key. In the age of uploading, cloud storage, and the electronic medical record, photographs in healthcare settings can now be readily sent to the medical record and sit behind a virtual firewall, safeguarding confidential patient health information. Current patient attitudes towards photography in clinic visits largely favor the use of it, particularly when the security of the photos within an EMR is addressed [8, 9]. The qualitative nature of dermatologic photographs, and dermatology being a largely visual discipline, highlight the potential for increased communication and patient‐provider engagement for shared decision‐making.

In addition to making patients more comfortable and motivated with continuing their treatments, the use of standardized photography can potentially improve care provided by dermatologists. A study by Buontempo et al. aimed to assess what minimum degree of hair loss is necessary before it is identifiable by a majority of dermatologists. They found a reduction of 22.66% of hair on standardized trichogram photographs is necessary for 75% of dermatologists to detect changes [10]. The same principle may apply to clinical photos using cameras or smartphones, whereas standardized photos can lead to quicker identification and treatment of hair loss before hair density is significantly reduced.

Currently, there is no standardized way of taking clinical photos of patients with hair loss. The patterns of hair loss in different conditions can vary significantly, furthering the importance of standardized and comprehensive photographs. Although pictures are not a treatment, we highlight here the use of standardized photographs for the management of hair loss disorders in an academic hospital where the current staff did not have formal picture‐taking training. This study aimed to standardize dermatologic photos consisting of pictures of the frontal, bilateral temporal, and crown of the scalp. This study looked at whether increasing awareness for the standardization of hair loss photos via educational resources would increase the percentage of patients being treated for hair loss who have clinical photos.

## 2 Root Cause Analysis

**FIGURE 1:**
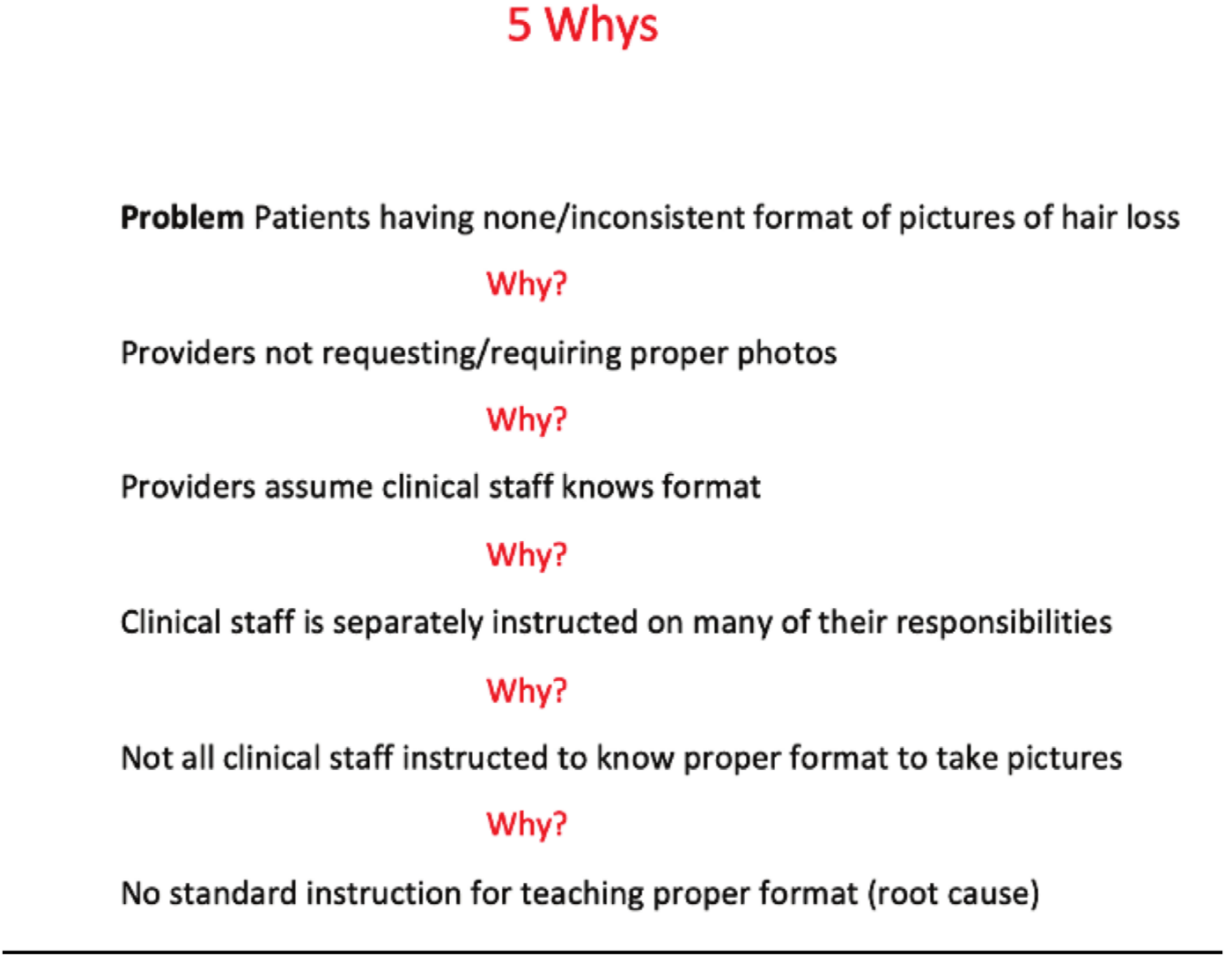
Root cause analysis utilizing the “5 Whys” method to evaluate why patients may not have standardized hair loss photographs.

**FIGURE 2:**
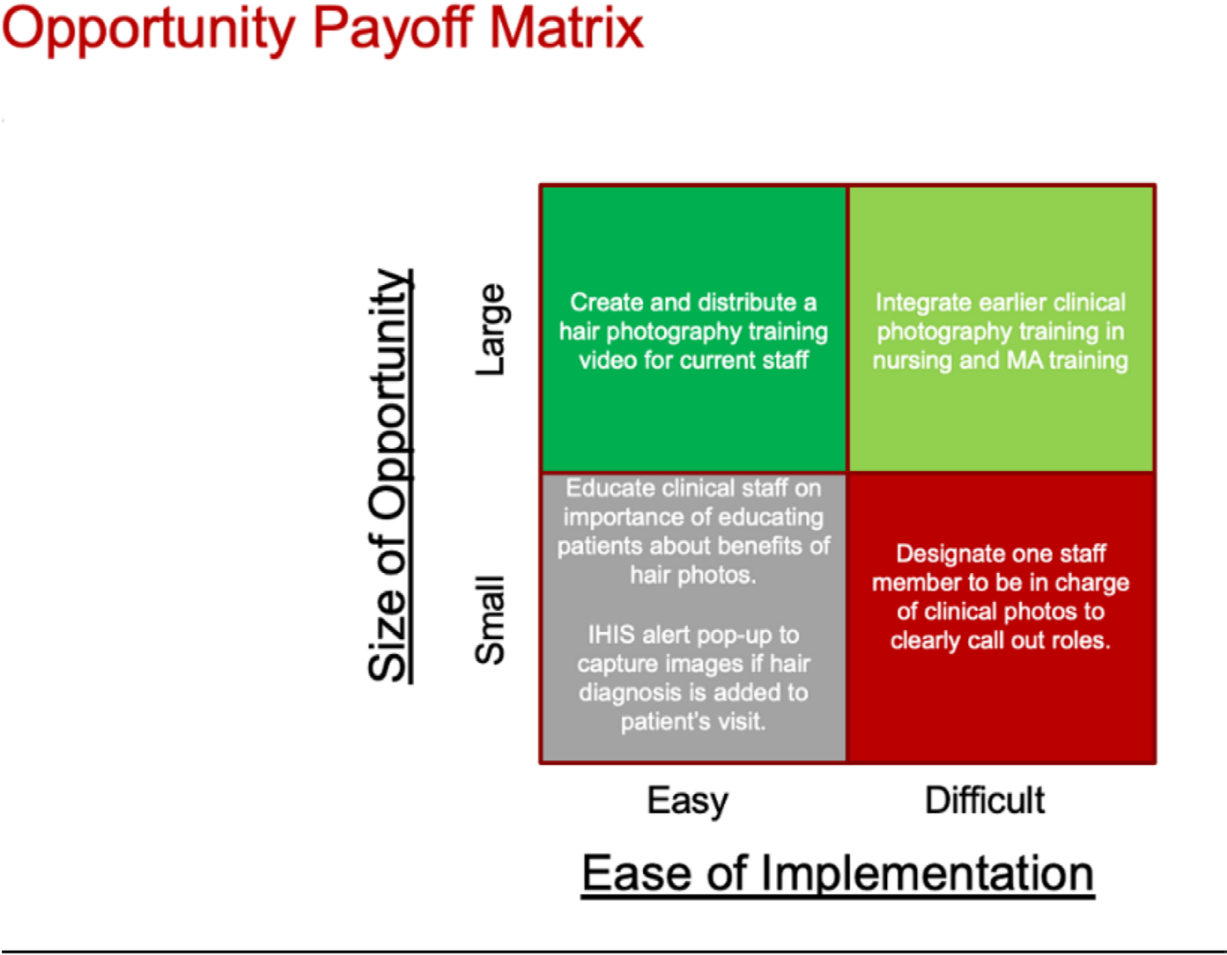
Interventions considered when evaluating implementation difficulty against potential impact.

We considered several underlying factors that could explain why proper, standardized hair loss photos were not taken during every patient visit related to hair loss (Figure 1). Other factors we considered included lack of time with the usual fast pace of dermatology clinics, limited availability of equipment for picture taking, and degree of patient willingness to have pictures taken. This study chose an opportunity payoff matrix to determine which intervention could have a big impact without difficult implementation (Figure 2). Educating clinical staff to educate patients about the benefits of clinical photos, and electronic medical record alerts about taking photos would be very easy to implement. However, because patient refusal is noted infrequently in charts and many providers have alert fatigue, these may not result in very many additional photos captured. Designating a staff member to be in charge of clinical photos may have some benefit and help solidify a well‐defined responsibility to ensure the photos are taken. Staff may push back on this extra work, however, and would still face the same issue with lack of training. In a large academic practice with several dermatologists seeing hair loss patients, assigning staff to be in charge of clinical photos is impractical. While earlier curricular changes could make a big impact, this would take extensive work and would be difficult to measure. However, videos as part of employee training have many benefits, such as increased interactivity, applicability to a diverse group of employees (via subtitles, etc.), flexibility, and better retention.

Thus, a training video to be created and disseminated would be a relatively simple intervention (1‐ to 2‐min video) that could immediately make a big impact in addressing the training deficit nurses and medical assistants describe. Ultimately, we chose to address the lack of training that nurses and medical assistants in the department of dermatology receive. We theorized that addressing the lack of training with an intervention could provide the most direct and objective benefit to the quality of hair loss assessments, be easily streamlined into clinic workflow, and ultimately improve patient care and treatment outcomes.

A survey given to the current nursing and medical assistant staff at the Ohio State University Wexner Medical Center Department of Dermatology (*n* = 7) highlighted that 75% of staff (*n* = 5) had not obtained formal picture training. In addition, a survey given to current dermatologists at the Ohio State University Wexner Medical Center Department of Dermatology found that only 67% of dermatologists reported ever training their nursing staff to take hair loss pictures. Interestingly, 100% of the staff were interested in participating in a formal training.

## 3 Video Implementation

**FIGURE 3:**
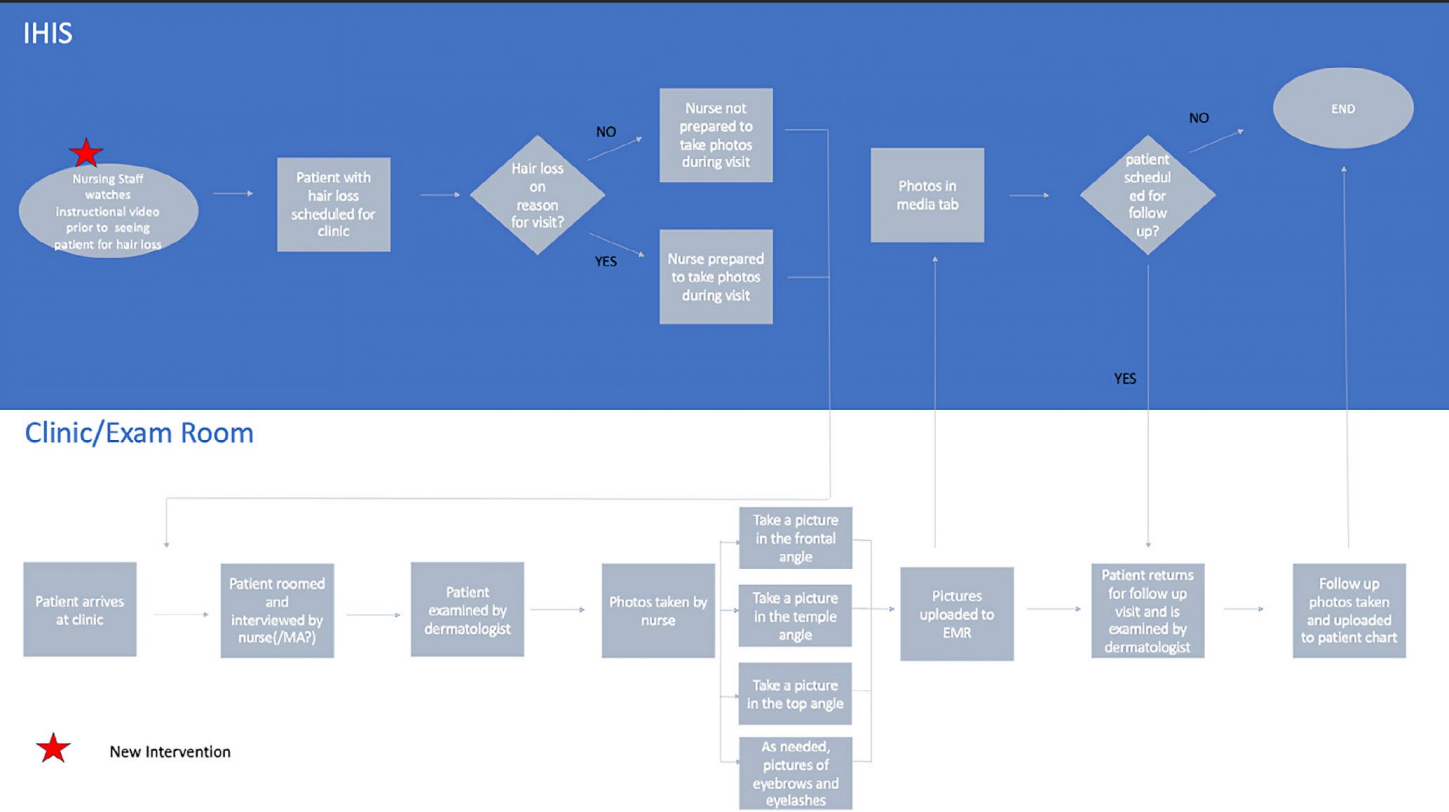
Flow chart depicting how the intervention was incorporated into clinic workflow.

We created a 2‐min video (Video S1) uploaded to YouTube that taught nurses and medical assistants how to take clinical pictures of hair loss in new and returning patients. This video aligned with the clinic workflow, so that pictures were taken after the patient had been examined by a dermatologist (Figure 3). We chose to utilize a video format for this training due to the ability to distribute a video asynchronously to the current nurses and medical assistants in the department for this study, and the potential to use the video for new hires as part of their clinic onboarding process as opposed to in‐person training, which may not be implemented consistently. A YouTube video also provides a convenient way for nurses and medical assistants to have a refresher on the topic when necessary.

**FIGURE 4:**
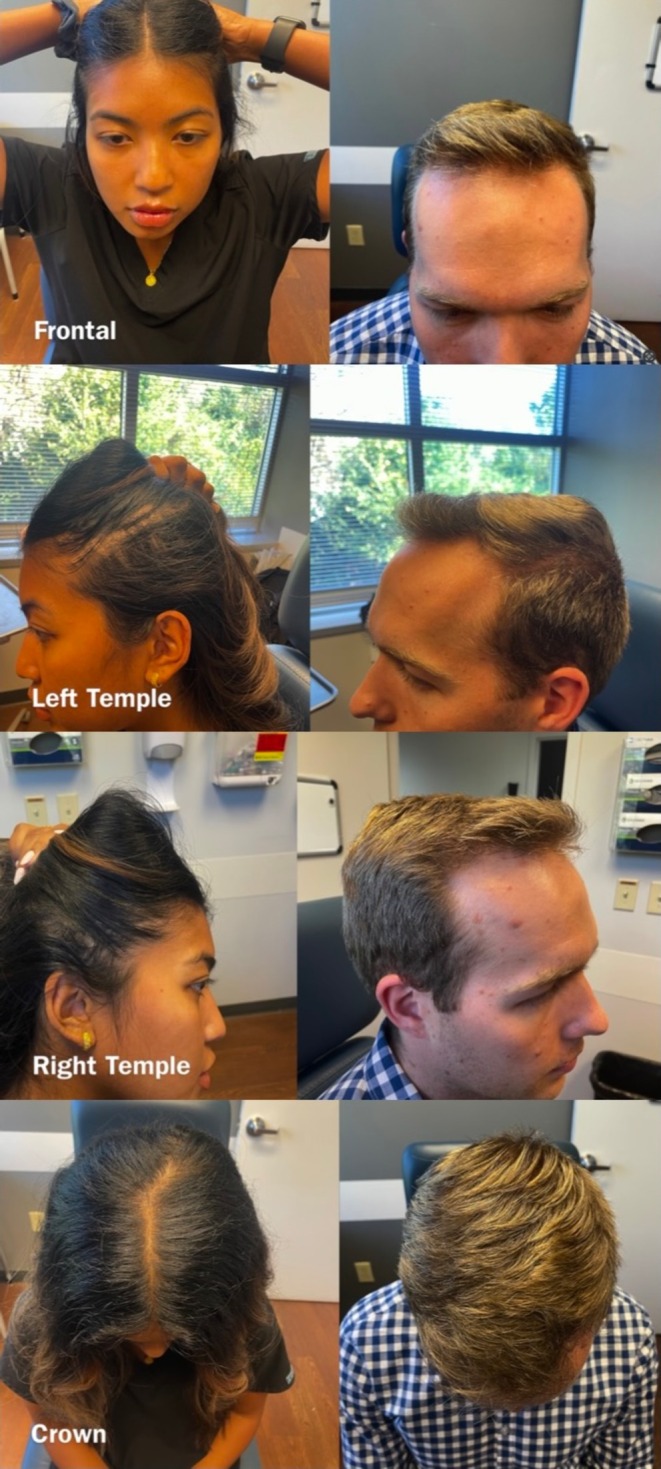
Depiction of standardized angles for hair loss photographs (Frontal, Bitemporal, Crown).

In the video, we reviewed foundational topics such as: how to use the EMR‐associated mobile application to take pictures, how to position patients in the examination room, how to take pictures from a minimum of three views—frontal, temporal, and top or crown (Figure 4), and how to upload pictures into patient charts.

We then distributed the educational video via email to all dermatology faculty and staff at the Ohio State University Wexner Medical Center. We regularly monitored viewership of the educational material via a survey distributed to the same faculty and staff.

## 4 Methods

**FIGURE 5:**
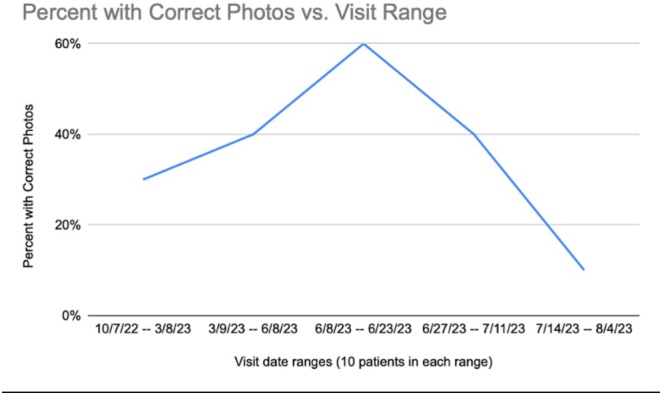
Percentage of patients with correctly documented photographs 1 year before the intervention.

To assess change from our intervention, we analyzed 50 (10%) randomly selected charts from a cohort of 500 unique patients seen for hair loss between October 2022 and August 2023 (Figure 5). Post‐intervention, we analyzed an additional 50 (10%) randomly selected charts from a separate cohort of 500 unique patients between October 2023 and January 2024. Only the photographs from one unique visit per patient were included to avoid duplication and ensure consistent sampling. Within this time frame, there were approximately 3500 unique visits for a primary diagnosis of alopecia.

Two researchers independently evaluated the quality of each photo to reduce bias. Inter‐rater reliability was not formally measured; however, a protocol was in place so that a third reviewer provided adjudication in the case of disagreement. This was utilized for one set of images during the review process. No additional patient data (including demographics) was collected outside of hair loss photographs.

## 5 Results

**FIGURE 6:**
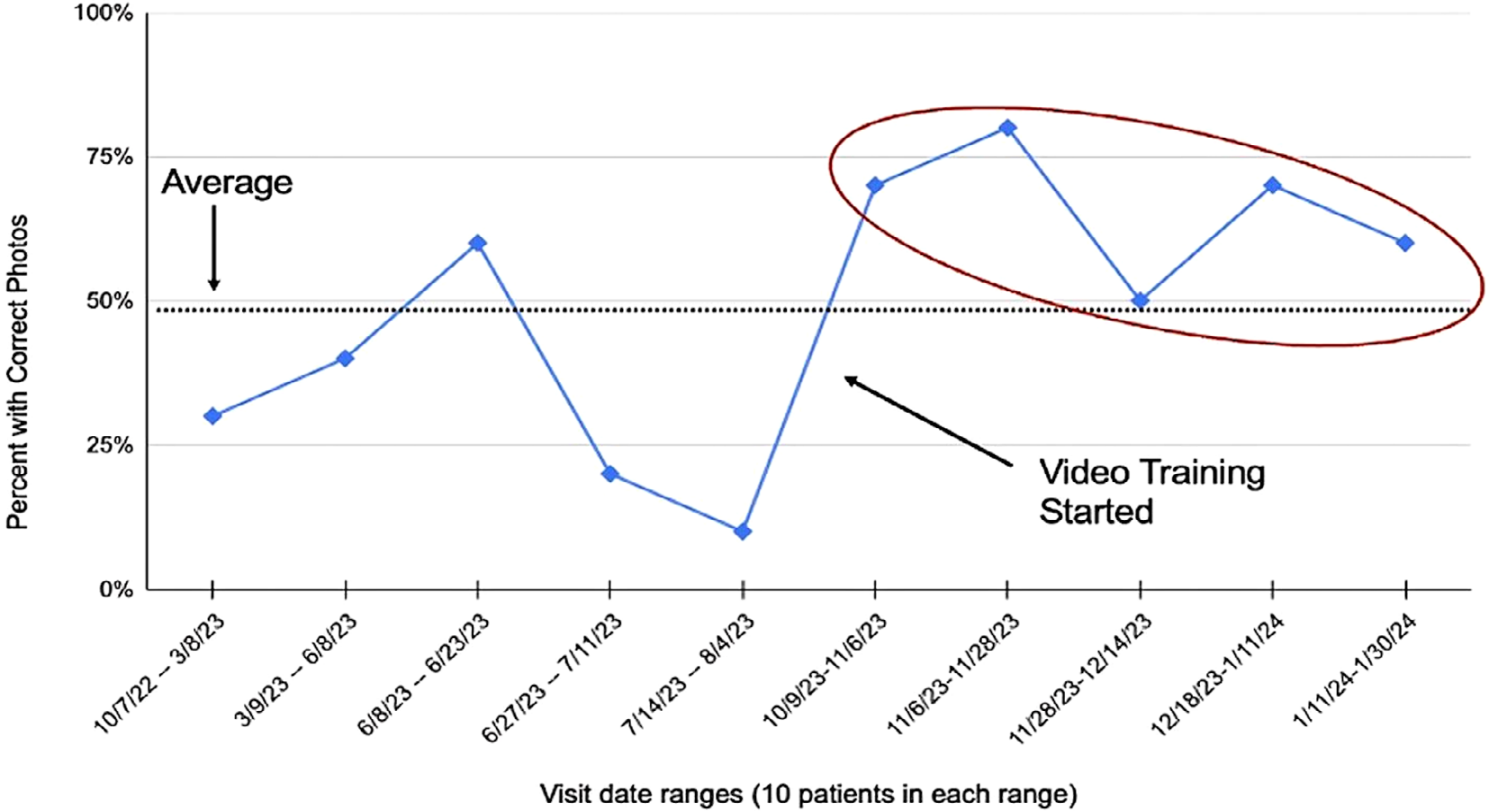
Average percent of correctly taken photographs following the intervention (up to 66%).

**FIGURE 7:**
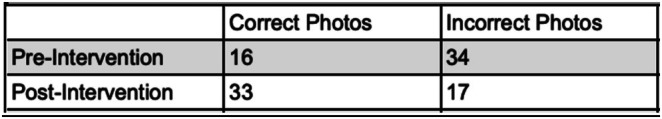
Number of correct photos versus incorrect photos taken pre‐ and post‐intervention.

The implementation of a training video demonstrating proper techniques for documenting hair loss pictures resulted in a statistically significant (*p* < 0.005) increase in correct picture documentation by nurses and medical assistants. Prior to the introduction of the training video, only 32% of hair loss patients had their pictures properly documented with the correct angles and number of minimum photographs. However, post‐training video dissemination, this figure dramatically increased to 66%, showcasing a substantial improvement in documentation practices (Figures 6 and 7). Using Chi‐square to analyze our data, there was significant improvement in the number of patients who had photos correctly taken (*p*‐value of 0.0007). The effect size by Cramer's *V* is 0.34, which is a medium effect size.

## 6 Limitations

Our video was specifically designed for nurses and medical assistants, which meant that the content in the video does assume a certain degree of technological literacy. Our video assumed that nurses and medical assistants have access to and understand how to log in to the mobile application. However, in our video, we demonstrated how to take the pictures and upload the pictures into the correct chart. Before watching our video, nurses and medical assistants only needed to understand how to log in to the mobile application.

Additionally, with our video intending to be a short demonstration of standardized angles for taking hair photos, we did not include a depiction of different hair types in our video or standardization of imaging techniques (e.g., lighting and camera settings). It is very important to consider different hair types to represent a diverse patient population and point out different considerations with various types of hair. For example, a lack of contrast between light hair and fair skin can make obtaining pictures more difficult, and hair with different textures or styles (i.e., cornrows, curly hair) may need to be positioned differently to ensure accurate photography [2]. Although we did not acknowledge this in our video, patients are encouraged to wear their hair as natural as possible to their visit to optimize the quality of photo documentation, and hair is positioned accordingly to obtain the desired angles.

## 7 Conclusion

This study demonstrates the positive impact of utilizing a training video to educate nurses and medical assistants on proper hair loss picture documentation techniques. By providing staff with accessible educational resources, healthcare facilities can effectively enhance picture documentation practices, with potential for improved dermatologic patient care and outcomes. The significant increase in both the quantity and quality of documented pictures highlights the value of having a targeted educational intervention to promote standardized documentation practices.

## 8 Future Directions

Moving forward, continued efforts should be made to reinforce the importance of consistent and accurate picture documentation among healthcare providers. This includes integrating the instructional YouTube video into onboarding sessions with every new team member that may be responsible for picture taking. Additionally, ongoing training and education initiatives should be implemented to ensure sustained adherence to documentation protocols. By prioritizing the development of staff skill set and knowledge in picture documentation, healthcare facilities can optimize patient care delivery and contribute to enhanced clinical decision‐making in the management of hair loss patients.

While follow‐up studies to assess sustained improvement have not been conducted, they represent an important next step. Future studies should assess long‐term improvements in standardized photographs. They should also look at patient satisfaction and comfort with photo documentation, as well as patient adherence to visits and treatments with improved documentation. Additionally, studies should be done to assess the quality and effectiveness of standardized photography across diverse hair types and textures to promote protocols that are equitable and applicable to all patients.

Lastly, new and emerging AI technology utilizes trichoscopic images to assess hair density and diameter. While this technology allows for more objective measures of hair regrowth or change, it can be time‐consuming and expensive to add to clinical practice, limiting practical use in a busy dermatologic clinic. Point‐of‐care photography, as described here, is comparatively cheap and fast to implement. Future studies may explore ways to integrate advanced imaging technology into clinical practice that are feasible for workflow, potentially combining this with standardized imaging to optimize patient care.

## Author Contributions

L.R., S.A., M.G., K.L., T.W., and B.D. contributed to project conception and design. L.R., A.K., S.R., and S.A. wrote the initial draft of the manuscript. All authors approved the final manuscript.

## Conflicts of Interest

Dr. Brittany Dulmage is a consultant for Novocure and serves on the Data Safety and Management Board of Hoth Therapeutics.

## Supporting information


**Video S1:** Instructional video depicting standardized angles for hair loss photography and the process of uploading images to a patient's medical record.

## Data Availability

Data sharing is not applicable to this article as no new data were created or analyzed in this study.
